# Religious Equality in Institutions

**Published:** 1907-11-09

**Authors:** 


### Nursing Administration.

### RELIGIOUS EQUALITY IN INSTITUTIONS.

Institution life has no room for religious intolerance. There are, it is true, establishments founded and endowed, or wholly supported, by members of particular churches, which maintain, and justly maintain, a certain uniformity of religious belief. It would be unfair to accuse of intolerance the heads of such institutions when they select members of the staff from persons of similar tenets. An orphanage distinctly Anglican and supported solely by members of the English Church would not appoint teachers of another communion. Nor would a Roman Catholic home for the dying desire to appoint nurses out of sympathy with the work carried on in its walls. The number of purely religious establishments is not, however, very large. For the most part institutions appealing for public support are intended for the relief of persons of all religious convictions, and the subscribers are very far from desiring to limit the choice of the governing committees to members of any particular religious denomination when vacant posts are to be filled. In all public institutions the principle has long been openly acknowledged of absolute religious equality, and the imposition of a religious test of any description is rightly regarded as an offence against the liberty of the staff. Complaints are, however, not infrequent of violations of this good rule by boards of guardians and hospital committees. The complaints are no doubt often unfounded, for rejected candidates are always prone to discover remote reasons for the disconcerting circumstance that another has been preferred to themselves. Yet so long as religious convictions continue to be held with burning force, and long may that be, certain good people will allow their choice to be biassed by the reflection that some candidates are, in their judgment, in the way of salvation and others not, or at any rate not so clearly. The only way in which to be quite sure of absolute impartiality in this particular is to require no information touching religious belief from the candidates. When it is a question of appointing a chaplain to minister to the spiritual needs of the sick the case is different. With regard to a nurse called solely to minister to their bodily needs, the committee is concerned solely with her qualifications as nurse and as woman. The nurse who recognises the authority of the Bishop of Rome in matters spiritual will not for that reason yield a less ready obedience to her matron in matters temporal. The Unitarian who but lightly esteems, may be, the decisions of the bishops touching a deceased wife's sister, is not for that reason less likely to uphold the best traditions of her training school. Nay, even those whose religious convictions are weak to breaking point may find in the wards a religion of service, and learn to worship an unknown God through their love for suffering humanity. Hence, we deprecate the introduction into forms supplied to candidates for vacant posts, of any invitation to them to make declaration of belonging to any particular religious body. Experience has shown that such declarations are readily filled in according to what it is supposed will be most acceptable to the authorities by such as attach no value to them, and it is wiser to leave no loophole through which aggressive people may profess to discern the relics of intolerance.

But religious equality does not mean religious indifference. Within the walls of the institution there should be not merely cold tolerance for diversity of opinions, but cordial opportunity for the exercise of religious obligations. It is easy to declare that no religious distinctions are made in selecting the staff, and yet to make the position of any who do not conform absolutely untenable. It is as intolerant to place obstacles in the way of attending early services as it is to insist on attendance at a given church or chapel. It is in the power of every matron to facilitate or impede the religious observances of her staff. She may establish clear cut rules which take all the heart out of her nurses' religion, or she may display a wise and kindly desire that all shall have an equal opportunity for worship in the manner most acceptable to them by habit or principles. True religious equality implies honour to every creed, but if every visit to a place of worship not approved by the authorities gives occasion for an icy look, then under whatever easy rule of faith the institution is governed, religious intolerance is rampant. To be truly tolerant, which it must never be forgotten includes being tolerant also of intolerance, is not an easy matter. Like every other virtue worth having, it is won by long practice. Prejudices are very easily acquired, and while one unsatisfactory member of a particular religious community will suffice for most people to start a prejudice against others holding similar opinions, two successive failures would carry most of us to a certainty that none of this kind were ever to be trusted. But the superintendent who looks below the surface will not lightly judge her nurses save by the evidences of character she is able to discern in their work, and she will be for ever on her guard against allowing herself to be biassed by distinctions of creed or habits of religious observance.

It is the essence of religious equality that religion should come to be set very high in the institution. When all are free to worship without let or hindrance so far as the claims of the daily routine admit, there will be room for that sane and balanced religious enthusiasm which is of the utmost value to all within the walls. It is the flaw in nursing considered as a force in moulding character that it concentrates the attention too much on narrowing details, and sometimes blots out from the soul the sense of the infinite background on which the scene is painted. That all workers should be lifted through the performance of their several duties to the clearest possible perception of the unseen is the aim of the best administrator. To achieve this aim the only road is liberty.

